# Non-Tobacco Nicotine dependence associated with increased Postoperative Complications following Intramedullary Nailing for Intertrochanteric Femur Fractures

**Published:** 2024

**Authors:** Sabrina M Pescatore, Sterling J DeShazo, Robert W Lindeman

**Affiliations:** 1 John Sealy School of Medicine, University of Texas Medical Branch, Galveston, Texas, USA; 2 Department of Orthopedic Surgery and Rehabilitation, University of Texas Medical Branch, Galveston, Texas, USA

**Keywords:** Intertrochanteric Fractures, Intramedullary Fracture Fixation, Intramedullary Nailing, Nicotine Dependence, Non-Tobacco Products, Postoperative Complications, Risk Factors

## Abstract

**Objectives:**

Nicotine usage is known to increase postoperative complications; however, studies show that the general population believes that non-tobacco nicotine delivery devices are a safer option compared to tobacco-based nicotine products. This study evaluates postoperative complications between non-tobacco nicotine dependent and non-nicotine dependent patients for intramedullary nailing in intertrochanteric femur fractures.

**Methods:**

Utilizing the TriNetX database, we retrospectively examined postoperative outcomes in patients aged 18 to 90 who underwent intramedullary nailing for intertrochanteric femur fractures between November 21, 2013, and November 21, 2023. Two cohorts were analyzed: Cohort A comprised nicotine-dependent patients without tobacco product usage (e.g. cigarettes or chewing tobacco) and Cohort B consisted of patients without any nicotine dependence. Propensity matching was performed for BMI, type 2 diabetes, alcohol/substance abuse, socioeconomic status, and demographic factors. Outcomes assessed included mortality, sepsis, pneumonia, revision, dehiscence, pulmonary embolism, nonunion, and deep vein thrombosis within 1 day to 6 months post-treatment.

**Results:**

A total of 2,041 non-tobacco nicotine dependent patients were matched with 22,872 non-nicotine dependent patients. Non-tobacco nicotine dependent patients experienced higher associated risk for numerous postoperative complications compared to non-nicotine dependent patients including increased risk for mortality within 6 months postoperatively (RR 1.386, 10.7% vs 7.7%, *P* = 0.001), sepsis (RR 1.459, 4.4% vs 3.0%, *P* = 0.027), and pneumonia (RR 1.751, 5.8% vs 3.3%, *P* = 0.001).

**Conclusions:**

Non-tobacco nicotine dependence increases postoperative complications for patients undergoing intramedullary nailing for intertrochanteric femur fractures. Our findings support the need for development of perioperative nicotine cessation guidelines for non-tobacco nicotine users.

**Level of evidence:**

Level III, Prognostic

## Introduction

Intertrochanteric femur fracture is a serious orthopedic condition that occurs between the greater trochanter and lesser trochanter of the femur. Regardless of treatment management, these fractures have high associations with morbidity and mortality, with a 20%–30% one year mortality rate in postoperative patients globally [1–5]. To help reduce the likelihood of these common postoperative complications, it is imperative to identify potentially modifiable risk factors in patients undergoing intertrochanteric fracture fixation.

Some risk factors are not modifiable for hip fracture patients. Osteoporosis and increased age have been strongly associated with intertrochanteric fractures, with more than 90% of these fracture types occurring in patients over 65 years old [6,7]. In patients below 65, intertrochanteric fractures are typically due to a high-energy mechanism such as a motor vehicle accident or falling from heights [8]. Other predisposing conditions in hip fractures include hypertension, hypothyroidism, Alzheimer’s disease, chronic obstructive pulmonary disease, psychiatric disorders, diabetes mellitus, and systemic arterial hypertension [9]. One well-defined and modifiable risk factor for both pre- and postoperative complications in hip fractures is smoking tobacco-based cigarettes or use of other tobacco-based products [10,11].

Nicotine from tobacco-based products has been linked to increased hip fracture healing time, increased instances of nonunion and malunion, predisposition of patients to infections, and increased likelihood of refracture [10–12]. To reduce the risk of postoperative complications in scheduled surgeries, tobacco-based smoking cessation remains the primary recommended perioperative treatment [13,14]. However, the impact of using non-tobacco nicotine delivery devices on the risk of postoperative complications in this context has not been investigated [15,16].

Usage of non-tobacco nicotine products such as e-cigarettes and vaporizers has risen markedly since the 2010–2012 timeframe [17,18]. Recent surveys indicate that the public views non-tobacco nicotine delivery devices as healthier alternatives to smoking tobacco-based cigarettes or using other tobacco-based products [19,20]. This perspective may lead hip fracture patients to believe that non-tobacco nicotine dependence does not pose a significant risk to their bone healing.

In this study, we analyzed a cohort of non-tobacco nicotine dependent patients versus controls with the aim of evaluating the effects of non-tobacco nicotine delivery on the risks of postoperative complications following intertrochanteric femur fractures. If significant risks exist, our results could contribute to development of effective preventive strategies with the goal of improving postoperative patient outcomes for fracture fixations.

## Materials and Methods

In this retrospective cohort analysis, data was retrieved from the TriNetX database, a global health collaborative research platform which provides access to de-identified electronic medical records. Because this study used only de-identified patient records and did not involve the collection, use, or transmittal of individually identifiable data, this study was exempted from Institutional Review Board approval.

We utilized the United States collaborative network within TriNetX, which comprises 40 healthcare organizations and over 93 million patients. We analyzed postoperative outcomes for patients between the ages of 18 to 90 years who had undergone intramedullary nailing for an intertrochanteric femur fracture from November 21, 2013, to November 21, 2023. Patients included in the study were identified using Current Procedural Terminology (CPT) and International Classification of Diseases, Version 10 (ICD-10) codes.

Two cohorts were evaluated for this study. Patients in Cohort A underwent intramedullary nailing (CPT:27245) for an intertrochanteric femur fracture (ICD10:S72.14) and had a dependence to nicotine (ICD10: F17.2) but not to tobacco cigarettes (ICD10:F17.21), chewing tobacco (ICD10:F17.220), any other tobacco products (F17.29), and had no tobacco usage identified (ICD10:Z72). Cohort B underwent intramedullary nailing for intertrochanteric femur fracture and did not have a dependence to nicotine, tobacco cigarettes, chewing tobacco, any other tobacco products, and had no tobacco usage identified.

To avoid demographic bias, cohorts were propensity matched for body mass index, type 2 diabetes (ICD10:E11), alcohol-related disorders (ICD10:F10), substance abuse disorders (ICD10:F11, F12, F13, F14, F115, F16, F18, F19), and low socioeconomic status (ICD10:Z59.1, Z59.6, Z59.7). Cohorts were also similarly matched for relevant demographic factors, such as age at event, ethnicity, race, and sex. The propensity score matching performed in this analysis was 1:1.

The outcomes evaluated were: mortality, sepsis (ICD10:A41, A41.9), pneumonia (ICD10:J18, J95.89), revision of intertrochanteric fracture intramedullary nailing (CPT:27245), wound disruption or dehiscence (ICD10:T81.3, T81.31, T81.31XA), pulmonary embolism (ICD10:I26, I26.99), nonunion (ICD10:S72.90KX, S72.90XP, S72.141P), and deep vein thrombosis (DVT) of the lower extremity (ICD10:I82.40, I82.439, I82.409, I82.41, I82.4Y, I82.4Z). All postoperative complications were analyzed between 1 day and 6 months following intramedullary nailing of intertrochanteric fractures. Outcomes were excluded if they occurred before or after the window indicated. Data is reported in risk ratios (RR), 95% confidence intervals (CI), and a risk comparison expressed as a *P* value.

### Source of funding:

This research was supported by the Institute for Translational Sciences at The University of Texas Medical Branch, supported in part by a Clinical and Translational Science Award (UL1 TR001439) from the National Center for Advancing Translational Sciences at the National Institutes of Health (NIH). The content is solely the responsibility of the authors and does not necessarily represent the official views of the NIH.

## Results

A total of 2,041 non-tobacco nicotine dependent patients (Cohort A) were matched with 22,872 non-nicotine dependent patients (Cohort B), with both cohort populations undergoing intramedullary nailing of intertrochanteric femur fractures. Before matching, non-tobacco nicotine dependent patients were an average of 64.9 years old ± 15.0 years. The cohort was 52.4% Male, 46.1% Female, and 1.5% Unknown. Most patients were White (80.4%), followed by African American (8.9%), Hispanic or Latino (5.1%), Asian (0.8%) and Unknown or Other Race (4.8%). The 22,872 non-nicotine dependent patients were an average age of 73.2 years old ± 13.1 years. The cohort was 35.4% Male, 61.5% Female, and 3.1% Unknown. Most patients were White (82.6%), followed by Hispanic or Latino (7.0%), African American (4.9%), Asian (2.3%) and Unknown or Other Race (3.2%).

Before propensity matching, Cohort A patients were significantly more likely to undergo intertrochanteric fracture nailing at a younger age (*P* < 0.001), to be Male (*P* < 0.001), and to be Black or African American (*P* < 0.001) (Table 1). Patients in Cohort A were also less likely to be Female (*P* < 0.001), White (*P* = 0.005), Hispanic or Latino (*P* = 0.001), or Asian (*P* < 0.001) (Table 2). After propensity matching, there were no significant demographic differences between Cohort A and Cohort B (Table 2).

**Figure 1:**
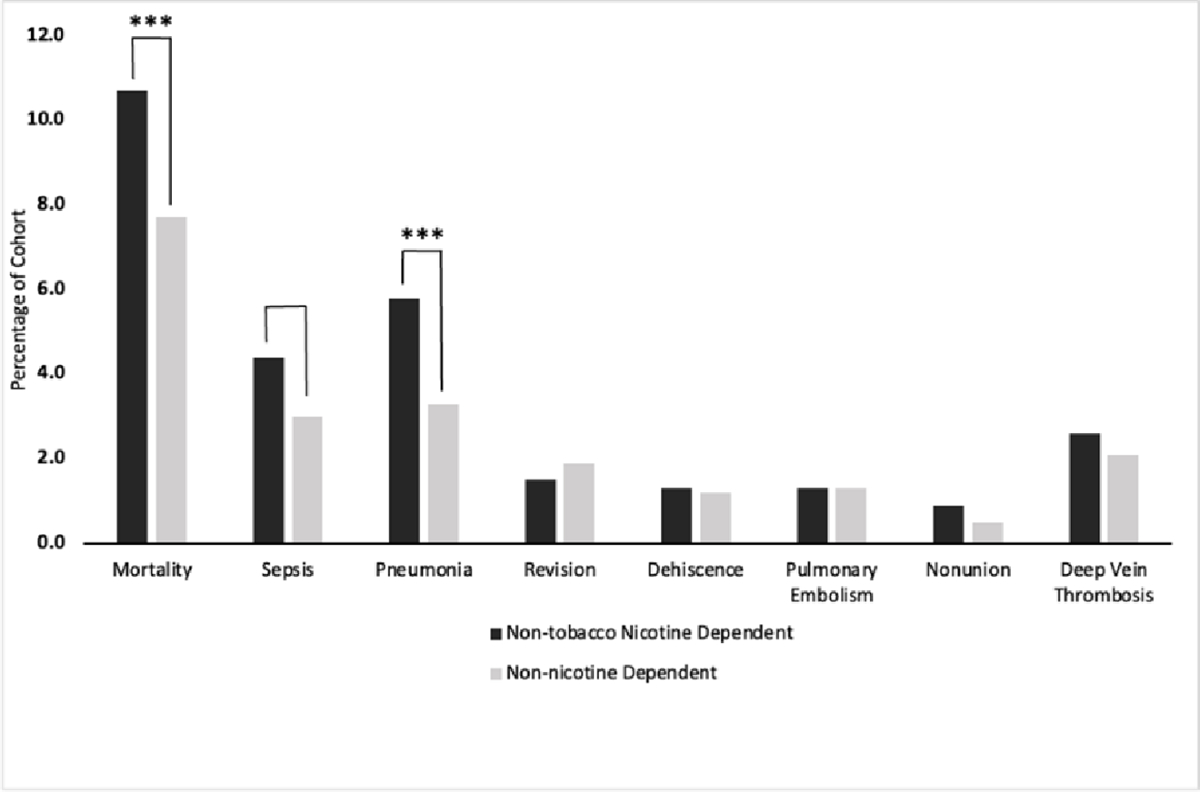
Risk represented as percent of population affected after propensity matching. Black indicates non-tobacco nicotine-dependent patients. Gray indicates non-nicotine-dependent patients. One asterisk indicates (P < 0.05), two asterisks indicate (P < 0.01), three asterisks indicate (P < 0.001).

Before propensity matching, patients in Cohort A were significantly more likely to die within 6 months of the procedure (*P* = 0.050) and develop pneumonia (*P* < 0.0001) or nonunion (*P* = 0.0001) (Table 3). After propensity matching, Cohort A experienced significantly higher postoperative complication risks for mortality within 6 months postoperatively (RR 1.386, 10.7% vs 7.7%, *P* = 0.001), sepsis (RR 1.459, 4.4% vs 3.0%, *P* = 0.027), and pneumonia (RR 1.751, 5.8% vs 3.3%, *P* = 0.001) (Table 4, Figure 1). Although not statistically significant, patients in Cohort A experienced nonunion at a higher incidence compared to Cohort B (17 vs. 11, RR 1.708, 0.9% vs. 0.5%, *P* = 0.173).

## Discussion

Though substantial research assessing the effects of newer non-tobacco nicotine delivery devices is lacking, the detrimental effects of tobacco-based smoking and nicotine ingestion have been extensively studied. Research has identified a link between tobacco-based smoking and higher mortality rates following hip fractures, although the role of nicotine alone has not been investigated [15,16,21]. However, nicotine has been shown to increase the risk of mortality and vascular respiratory events for patients undergoing major surgery [22–24]. Our results show a significant increase in mortality risk within 6 months of intertrochanteric fracture intramedullary nailing in patients who were non-tobacco nicotine dependent, compared to controls.

Our results demonstrated a significant increase in sepsis or pneumonia risk for non-tobacco nicotine dependent patients, compared to controls. Nicotine has previously been established as a contributor to increasing the cellular oxidative stress burden as well as the production of inflammatory markers such as c-reactive protein, soluble intercellular adhesion molecule, and the danger signal machinery high-mobility group box 1 (HMGB1) [25–27]. The coordination of key inflammatory processes, the augmented state of inflammation, or vascular involvement of nicotine may potentially play a role in the pathogenesis of postoperative sepsis or pneumonia [7,28,29].

Recent data suggests that nicotine alone can decrease bone remodeling effects of osteoblasts and osteoclasts, further identifying nicotine use and dependence as a potential risk factor for bone healing and remodeling [27,29]. This postoperative complication was not observed following propensity matching in our study. Non-tobacco nicotine dependent patients did experience higher instances of nonunion following intramedullary nailing for intertrochanteric fractures in our study (17 compared to 10 patients), but the results were not statistically significant. In addition, tobacco-based cigarette smoking has been linked to postoperative surgical revision, dehiscence, pulmonary embolism, and DVT’s [22]. These complications were also not observed following propensity matching in our study. It is likely that the sample size of our population limited adequate analysis of these additional variables. Future studies should focus on the effects of nicotine on revision, nonunion, dehiscence, pulmonary embolism, and DVT’s, as these complications pose a significant risk to patient recovery.

To reduce the risk of postoperative complications for scheduled surgeries, perioperative tobacco-based smoking cessation remains an effective treatment and can stimulate long-term smoking cessation [13,14]. Current literature suggests that cessation within 4 weeks of medical procedures can markedly decrease complications [5]. Intervals of smoking cessation greater than 4 weeks have been indicated to be most effective in the reduction of complications [30]. However, there are limited studies on the effects of tobacco-based smoking cessation following acute fracture surgeries. Research demonstrates that smoking cessation subsequent to emergency fracture surgery can significantly decrease both the overall count of postoperative complications and the total number of complications [25]. Discontinuation of smoking also reduces the odds of developing a postoperative complication by over 2.5 times compared to those who continued smoking during the same timeframe [31].

Despite limited objective data, Ashour et al recommends that non-tobacco nicotine delivery devices such as e-cigarettes and vaporizers should be treated similarly to tobacco-based cigarettes during the perioperative period [28]. Preliminary data via in vitro studies and case reports suggest that non-tobacco nicotine delivery devices do pose a non-zero surgical risk for postoperative complications, including delayed wound healing, higher instances of nonunion, increased inflammation and cytotoxicity, and impaired immune response [7,28,32].

Our study identifies a significant relationship between non-tobacco nicotine dependent patients and increased postoperative risks following intramedullary nailing for intertrochanteric fractures. With limited in vivo studies available, our results highlight the need for further exploration of the mechanism by which these complications occur in the context of non-tobacco nicotine dependency.

### Limitations

This study has several limitations. The usage of administrative medical coding introduces the risk of misclassification or other administrative confounding in the data. To mitigate these errors, we implemented multiple outcome specific ICD10 codes that categorize the most commonly used codes when defining a particular outcome. In addition, specific ICD10 codes and propensity matching for all substance use disorders, low SES, and various health conditions were applied for exclusionary purposes to ensure proper cohort construction. However, the use of exclusionary codes and propensity matching for many variables may have limited our analyses by making our cohorts too small or by excluding valid patients. The timeframe we selected may not allow for adequate longitudinal follow-up in tracking nonunion and revision surgeries. We also could not confirm morphology of fractures for those coded as nonunion. Though the nature of our retrospective cohort analysis generalizes our results to a broader population, our findings may not be universally applicable. Utilizing a database allows for the limitation of confounding pathologies, however, it does not account for all patient-specific characteristics and limits our study controls.

## Conclusion

This study highlights the potential harmful effects non-tobacco nicotine dependence has on intertrochanteric fracture healing and suggests that it is not a safer option versus tobacco-based smoking, contrary to public opinion. Though we analyzed nicotine dependence postoperative complications in the context of acute fractures, these results may have broader implications. To the authors’ knowledge, no formal guidelines currently exist regarding the cessation of non-tobacco nicotine products prior to or following orthopedic operative procedures. Our findings may aid in the development of perioperative guidelines for non-tobacco nicotine dependent patients outside of the trauma setting. Clinical trials regarding the potential health hazards of nicotine in the context of perioperative cessation may better allow clinicians to optimize their surgical management protocol.

## Figures and Tables

**Table 1:** Cohort demographics before 1:1 propensity matching of (A) non-tobacco nicotine dependent vs. (B) non-nicotine dependent patients.

Cohort	Demographic	Mean +/− Standard Deviation	Patients	% of Cohort	*P*-Value	Standard Difference
A	Age at Index	64.9 +/− 15.0	2,041	100%	<0.001	0.588
B		73.2 +/− 13.1	22,872	100%		
A	White		1,635	80.10%	0.005	0.063
B			18,882	82.60%		
A	Female		940	46.10%	<0.001	0.314
B			14,074	61.50%		
A	Unknown Ethnicity		274	13.40%	0.71	0.009
B			3,004	13.10%		
A	Not Hispanic or Latino		1,663	81.50%	0.075	0.042
B			18,259	79.80%		
A	Hispanic or Latino		104	5.10%	0.001	0.081
B			1,609	7.00%		
A	Black or African American		184	9.00%	<0.001	0.161
B			1,128	4.90%		
A	Male		1,071	52.50%	<0.001	0.357
B			8,016	35.00%		
A	Other Race		25	1.20%	0.061	0.047
B			410	1.80%		
A	Asian		16	0.80%	<0.001	0.124
B			529	2.30%		

**Table 2:** Cohort demographics after 1:1 propensity matching of (A) non-tobacco nicotine dependent vs. (B) non-nicotine dependent patients.

Cohort	Demographic	Mean +/− Standard Deviation	Patients	% of Cohort	*P*-Value	Standard Difference
A	Age at Index	65.0 +/− 14.9	2,027	100%	0.156	0.045
B		64.4 +/− 16.0	2,027	100%		
A	White		1,629	80.40%	0.874	0.005
B			1,633	80.60%		
A	Female		935	46.10%	0.636	0.015
B			920	45.40%		
A	Unknown Ethnicity		270	13.30%	0.465	0.023
B			286	14.10%		
A	Not Hispanic or Latino		1,653	81.50%	0.778	0.009
B			1,646	81.20%		
A	Hispanic or Latino		104	5.10%	0.513	0.021
B			95	4.70%		
A	Black or African American		181	8.90%	0.913	0.003
B			183	9.00%		
A	Male		1,062	52.40%	0.637	0.015
B			1,077	53.10%		
A	Other Race		25	1.20%	0.112	0.05
B			15	0.70%		
A	Asian		16	0.80%	0.448	0.024
B			12	0.60%		

**Table 3:** Postoperative outcome evaluations before 1:1 propensity matching of non-tobacco nicotine dependent vs. non-nicotine dependent patients.

Outcome	Non-tobacco nicotine Dependent	Non-nicotine Dependent	Risk Ratio (Dependent: Non-dependent)	95% CI	*P*-value
Mortality	214 (10.8%)	2032 (9.5%)	1.143	(1.001, 1.306)	0.05
Sepsis	78 (4.5%)	777 (3.9%)	1.143	(0.910, 1.435)	0.252
Pneumonia	81 (5.5%)	664 (3.5%)	1.546	(1.235, 1.935)	<0.0001
Revision	29 (1.4%)	347 (1.6%)	0.908	(0.623, 1.323)	0.615
Dehiscence	25 (1.3%)	186 (0.9%)	1.483	(0.979, 2.246)	0.061
Pulmonary Embolism	24 (1.3%)	246 (1.2%)	1.077	(0.710, 1.634)	0.727
Nonunion	16 (0.8%)	68 (0.3%)	2.583	(1.501, 4.444)	<0.0001
Deep Vein Thrombosis	51 (2.7%)	504 (2.4%)	1.11	(0.835, 1.474)	0.473

**Table 4:** Postoperative outcome evaluations after 1:1 propensity matching of non-tobacco nicotine dependent vs. non-nicotine dependent patients.

Outcome	Non-tobacco nicotine Dependent	Non-nicotine Dependent	Risk Ratio (Dependent: Non-dependent)	95% CI	*P*-value
Mortality	214 (10.7%)	154 (7.7%)	1.386	(1.138, 1.689)	0.001
Sepsis	78 (4.4%)	56 (3.0%)	1.459	(1.042, 2.044)	0.027
Pneumonia	86 (5.8%)	57 (3.3%)	1.751	(1.262, 2.430)	0.001
Revision	31 (1.5%)	39 (1.9%)	0.795	(0.498, 1.269)	0.335
Dehiscence	25 (1.3%)	23 (1.2%)	1.099	(0.626, 1.929)	0.743
Pulmonary Embolism	25 (1.3%)	25 (1.3%)	1.019	(0.587, 1.767)	0.947
Nonunion	17 (0.9%)	10 (0.5%)	1.708	(0.784, 3.720)	0.173
Deep Vein Thrombosis	49 (2.6%)	40 (2.1%)	1.241	(0.821, 1.874)	0.305
